# Metastatic Pancreatic Cancer Is Dependent on Oncogenic Kras in Mice

**DOI:** 10.1371/journal.pone.0049707

**Published:** 2012-12-03

**Authors:** Meredith A. Collins, Jean-Christophe Brisset, Yaqing Zhang, Filip Bednar, Josette Pierre, Kevin A. Heist, Craig J. Galbán, Stefanie Galbán, Marina Pasca di Magliano

**Affiliations:** 1 Cellular and Molecular Biology Program, University of Michigan, Ann Arbor, Michigan, United States of America; 2 Department of Radiology, University of Michigan, Ann Arbor, Michigan, United States of America; 3 Center for Molecular Imaging, University of Michigan, Ann Arbor, Michigan, United States of America; 4 Department of Surgery, University of Michigan, Ann Arbor, Michigan, United States of America; 5 Department of Radiation Oncology, University of Michigan, Ann Arbor, Michigan, United States of America; 6 Cell and Developmental Biology, University of Michigan, Ann Arbor, Michigan, United States of America; 7 Comprehensive Cancer Center, University of Michigan, Ann Arbor, Michigan, United States of America; Technische Universität München, Germany

## Abstract

Pancreatic cancer is one of the deadliest human malignancies, and its prognosis has not improved over the past 40 years. Mouse models that spontaneously develop pancreatic adenocarcinoma and mimic the progression of the human disease are emerging as a new tool to investigate the basic biology of this disease and identify potential therapeutic targets. Here, we describe a new model of metastatic pancreatic adenocarcinoma based on pancreas-specific, inducible and reversible expression of an oncogenic form of Kras, together with pancreas-specific expression of a mutant form of the tumor suppressor p53. Using high-resolution magnetic resonance imaging to follow individual animals in longitudinal studies, we show that both primary and metastatic lesions depend on continuous Kras activity for their maintenance. However, re-activation of Kras* following prolonged inactivation leads to rapid tumor relapse, raising the concern that Kras*-resistance might eventually be acquired. Thus, our data identifies Kras* as a key oncogene in pancreatic cancer maintenance, but raises the possibility of acquired resistance should Kras inhibitors become available for use in pancreatic cancer.

## Introduction

Pancreatic ductal adenocarcinoma (PDA), the most common form of pancreatic cancer, is frequently associated with mutations of the Kras oncogene, most commonly KRAS^G12D^ [1], [2]. Mutations of the tumor suppressor p53 −most commonly R175H [3]− are frequently observed in human samples [1]. Expression of mutant Kras^G12D^ (Kras*) and mutant p53^R172H^ -the mouse ortholog of R175H- in the mouse pancreas was used to generate the KPC model. KPC mice closely mimic the progression of the human disease [4], [5] and respond to therapeutics in a similar manner as human patients. In contrast, tumors transplanted in immuno-compromised mice poorly predict therapeutic response [6], [7]. The KPC model is thus ideally suited to study pancreatic cancer formation. However, in this model mutant Kras expression is irreversible. Thus, KPC mice are not suitable to study the role of Kras* in tumor maintenance. Since drugs targeting Kras* are currently unavailable, genetic modeling of Kras inhibition is the only option to determine whether this oncogene is required for tumor maintenance.

We, and others, have recently described the inducible-Kras*p53^+/−^ (iKras*p53^+/−^) mouse model of pancreatic cancer, that allows tissue-specific, inducible and reversible expression of mutant Kras in combination with a loss of function allele of the tumor suppressor p53 [8], [9]. iKras*p53^+/−^ mice develop invasive, but non-metastatic pancreatic cancer that is dependent on sustained Kras* activity for its growth and maintenance. In other mouse models of pancreatic cancer, as well as in other tumor models, loss of function of p53 accelerated tumor formation but only infrequently gave rise to metastatic disease. In contrast, expression of mutant p53 has been shown to be highly pro-metastatic [10], [11], [12]. There is a certain variability in these findings: for instance, metastatic potential has been described by other groups using KC or iKras* mice combined with loss of function allele of p53 [9], [13], thus there might be additional effects to consider, such as genetic background of the mice. Given that in our mouse colony p53 loss-of function did not confer metastatic potential to iKras* mice, we generated iKras* mice that also carried a mutant p53 allele (p53^R172H^, hereby p53*). Our goal was to generate a metastatic model where we could address the role of Kras* in the maintenance of metastatic pancreatic cancer by following mice in longitudinal studies, using *in vivo* imaging.

**Figure 1:**
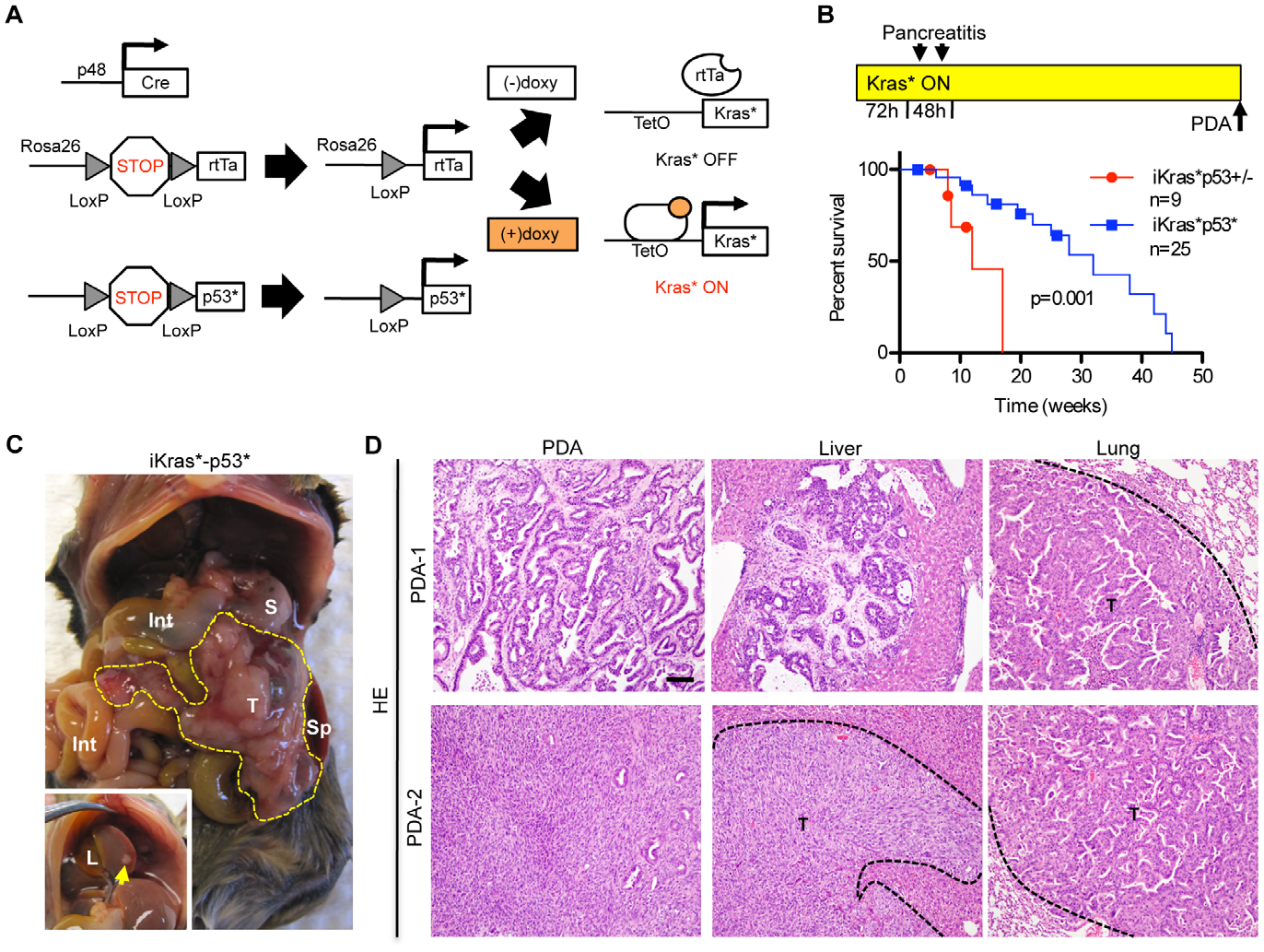
The iKras*p53* model of metastatic pancreatic adenocarcinoma. (**A**) Genetic makeup of iKras*p53* mice. (**B**) Experimental design: doxy was administered continuously, starting at weaning. Acute pancreatitis was induced within 72 hrs, then the animals were aged until they developed tumors. Kaplan-Meier survival curve. iKras*p53*, n = 25; iKras*p53^+/−^, n = 9. Log-rank statistical analysis yielded a P value of 0.001. (**C**) Gross morphology pictures of a primary tumor and liver metastases. T: tumor, S: stomach, Sp: spleen, Int: intestine, L: liver (**D**). Histology of a moderately differentiated (top row) and an un-differentiated (bottom row) pancreatic tumor; liver and lung metastases. T: tumor. Scale bar 100 um.

## Results

**Figure 2:**
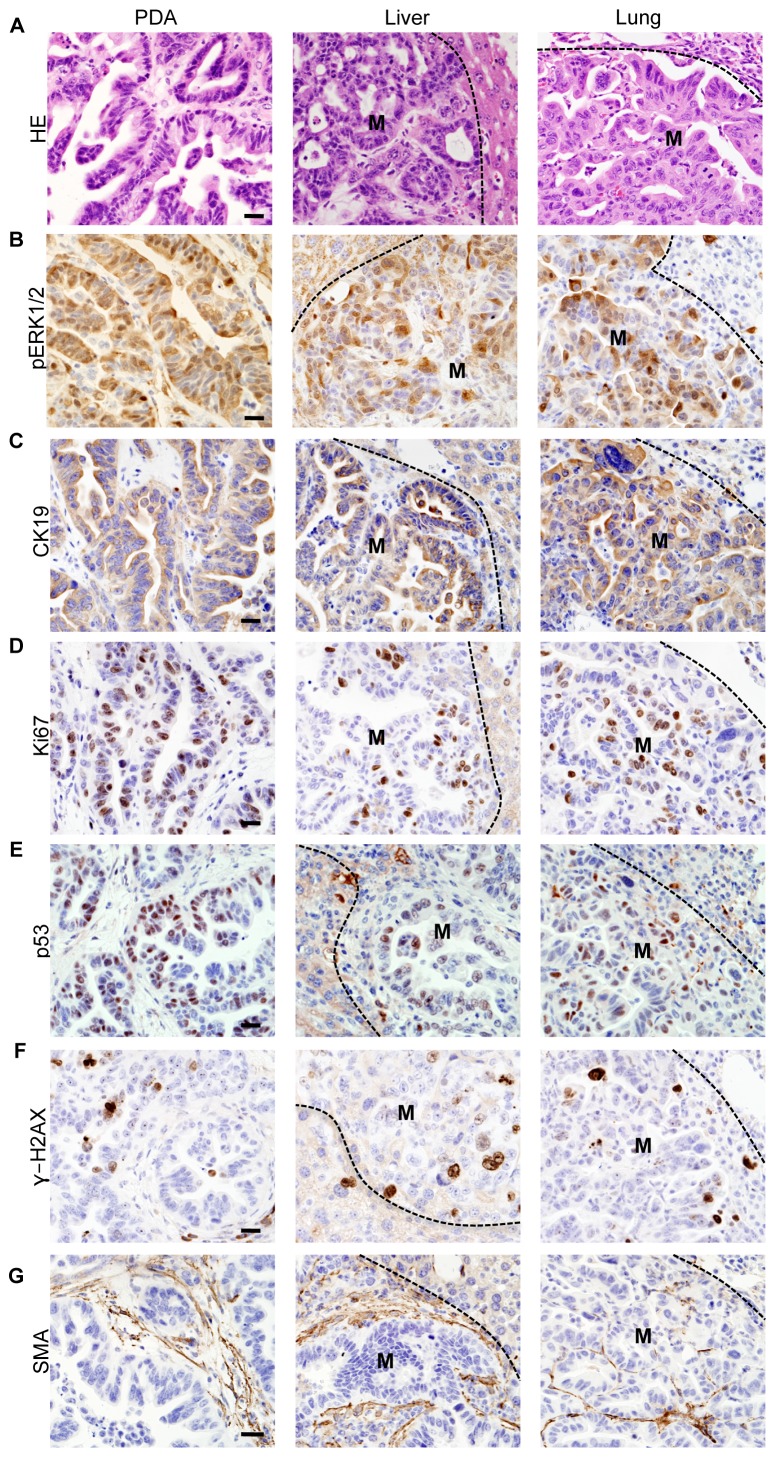
Characterization of iKras*p53* primary tumor and metastases. (**A**) Histology of a primary pancreatic adenocarcinoma and metastases to liver and lung. (**B–G**) Immunohistochemistry of primary tumor and metastases for: (**B**) phospho-ERK1/2; (**C**) CK19; (**D**) Ki67; (**E**) p53; (**F**) γH2AX; (**G**) αSMA. M: metastasis. Scale bar 20 um.

In iKras*p53* mice, the pancreas-specific p48-Cre (Ptf1a-Cre) [14] recombines a floxed stop cassette inserted in the Rosa26 locus, thus activating expression of the transcriptional activator rtTa [15]. Cre recombination also induces expression of p53^R172H^ (p53*) [16] from its endogenous locus upon recombination of a floxed stop cassette. The rtTa is transcriptionally active in the presence of doxycycline (doxy), and inactive in its absence. Thus, the TetO-Kras^G12D^ (Kras*) [17] allele can be transcribed in an inducible, tissue-specific and reversible manner by administering doxycycline to the animals’ water (**Fig. 1A**). In order to induce carcinogenesis, iKras*p53* mice were placed on doxy at weaning, followed by a short burst of acute pancreatitis to promote PanIN formation as previously described [18], [19]. The animals were then maintained on doxy until they developed PDA and had to be euthanized or succumbed, between 2 and 45 weeks following doxy administration (**Fig. 1B** **and** **Table 1**). Interestingly, survival of iKras*p53* animals was longer than that of iKras*p53^+/−^ mice (see Kaplan Meier curve in **Fig. 1B**); however, the reason for this difference remains unclear. At necropsy, iKras*p53* animals presented with a tumor mass frequently in the head of the pancreas, along with visible metastatic lesions (**Fig. 1C**). A subset of the animals also presented with hemorrhagic ascites (n = 5). The histology of the primary tumor revealed moderately to un-differentiated pancreatic adenocarcinoma with abundant desmoplastic stroma (**Fig. 1D** **,** **Fig. 2A** and **Table 1**) similar to what has been found in iKras*p53^+/−^ animals [8]. Metastatic lesions were highly prevalent in the liver (**Fig. 1C** inset, **1D,** **Fig. 2A** and **Table 1** **)**, and less frequent in the lungs; duodenal invasion was also occasionally observed (**Fig. 1D** **,** **Fig. 2A** **,** **Table 1** and data not shown**)**. Both primary tumors and metastases expressed phospho-ERK1/2, a downstream effector of Kras (**Fig. 2B**). Further characterization of the tumors and metastases revealed expression of PDA markers, such as CK19 (**Fig. 2C**), high proliferative index as measured by Ki67 staining (**Fig. 2D**), accumulation of mutant p53 protein (**Fig. 2E**), genomic instability as detected by γ-H2AX expression (**Fig. 2F**), and accumulation of desmoplastic stroma including smooth muscle actin-expressing fibroblasts (**Fig. 2G**). Thus, the iKras* p53* mouse model recapitulates the histology and biological behavior of human pancreatic cancer and previous mouse models with the additional ability to control Kras* expression in a time and organ-selective manner.

**Table 1:** Pathology of iKras*p53* mice.

ID	Survival(weeks)	Classification	Grade				Metastasis					Ascites
			W	M	P	U	LN	Duo	Spleen	Liver	Lung	
4067	**18**	PanIN III										
4292	**42**	PDA				**X**	Y	Y	N	Y	Y	–
4659	**25**	PDA			**X**		Y	Y	Y	–	–	Y
4668	**28**	PDA		**X**			Y	Y	N	Y	–	Y
5552	**32**	PDA		**X**			Y	N	N	N	N	Y
5820	**44**	PDA		**X**			N	N	N	–	–	–
5821	**19.5**	PDA			**X**		Y	Y	N	–	–	Y
5825	**48**	PDA		**X**			Y	N	N	–	–	–
5827	**45**	PDA		**X**			Y	N	N	Y	N	–
6106	**38**	PanIN III										
6649	**22**	PanIN III										
6977	**37**	PanIN II										
6989	**34**	PDA		**X**			N	N	N	–	–	–
7275	**42.5**	–										
7435	**6**	PDA		**X**			N	Y	N	–	–	Y
7994	**12**	PanIN II										–
8460	**14.5**	PDA			**X**		Y	Y	N	–	–	–
8461	**10**	–										
8847	**16**	PanIN II										
9261	**22**	PanIN II										
9805	**15**	PDA		**X**			Y	Y	N	Y	Y	–
9806	**15**	PanIN III										
11326	**2**	–										

**Histological Grade** - W: well differentiated; M: moderately differentiated; P: poorly differentiated; U: undifferentiated (sarcomatoid).

**Presence of Metastasis** - Y: yes; N: no; –: not available.

**Figure 3:**
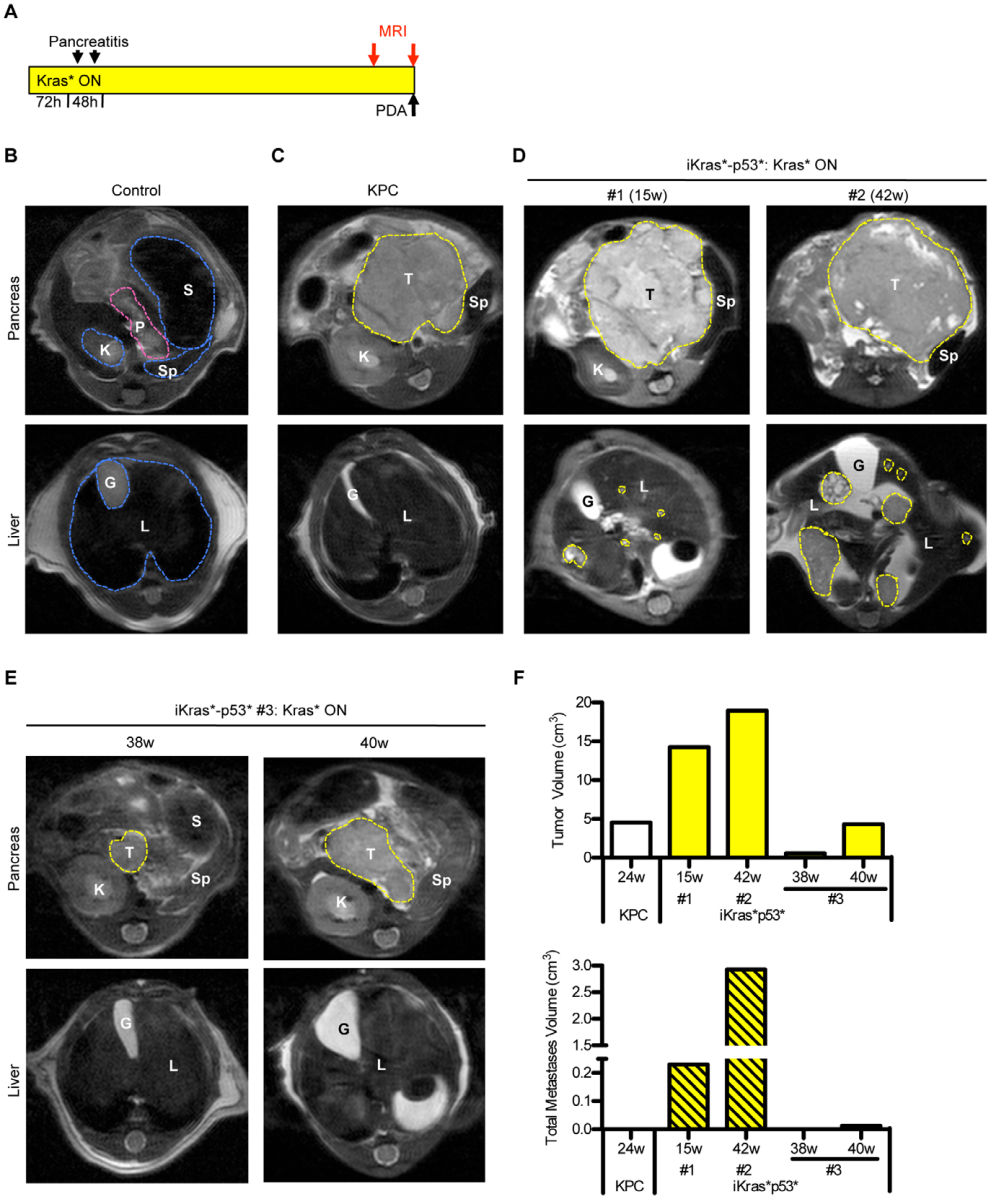
In vivo imaging of the pancreas, pancreatic tumors and metastases. (**A**) Experimental design. (**B**) MRI of a control mouse pancreas and liver. P: pancreas, S: stomach, Sp: spleen, K: kidney, L: liver, G: gallbladder. (**C**) Large pancreatic mass (T), but no metastatic lesions in a KPC mouse. (**D**) Two iKras*p53* mice on doxy 15 weeks (left) and 42 weeks (right) show a large pancreatic mass and liver metastases. (**E**) Identification of smaller tumors (iKras*p53* #3, left panel, 38 weeks) can be monitored as they develop into larger tumors (iKras*p53* #3, right panel, 40weeks). (**F**) Volume measurements of both primary tumors and combined metastases for individual KPC and iKras*p53* animals at the indicated time points.

**Figure 4:**
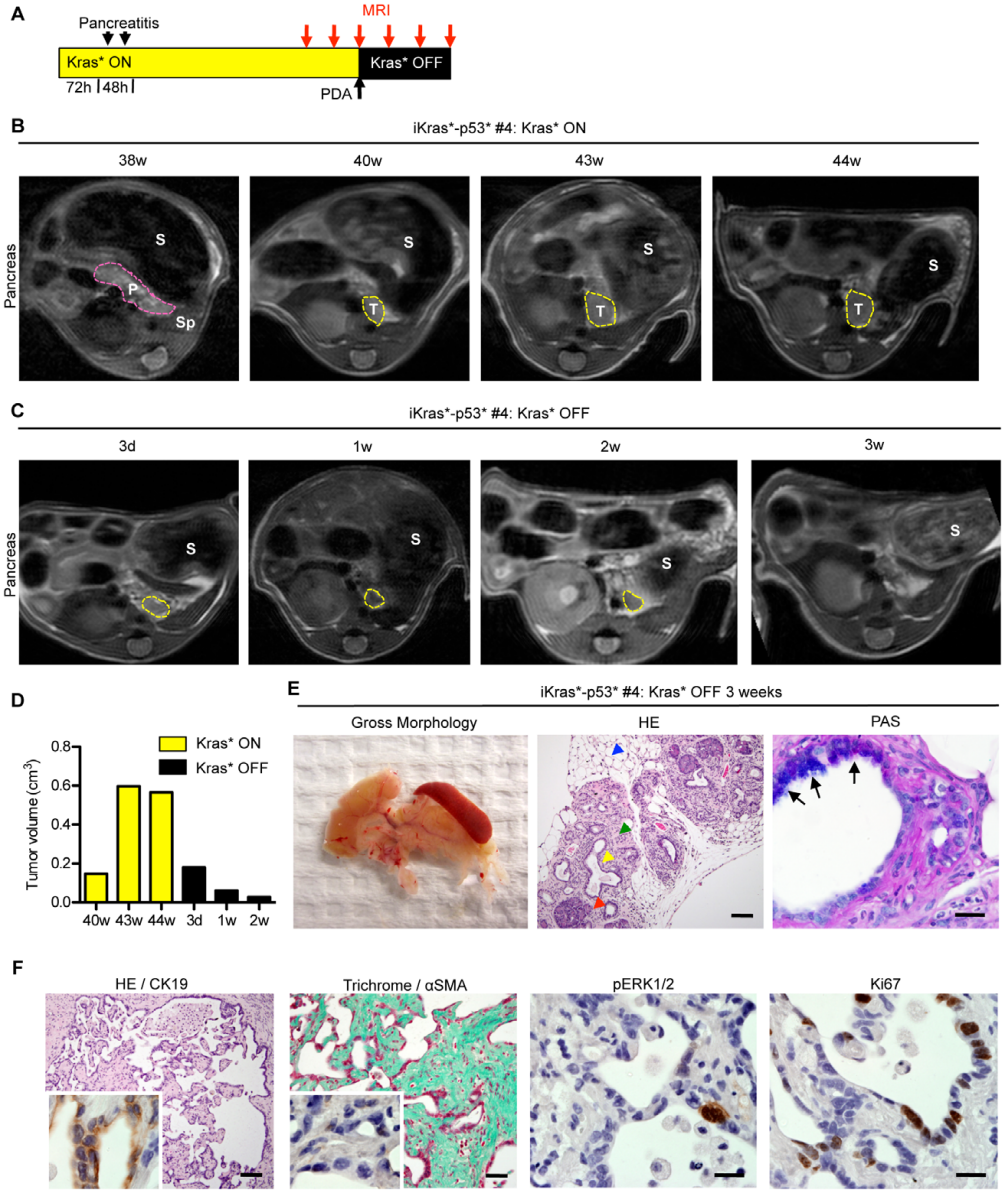
Longitudinal imaging of pancreatic tumor growth and regression. (**A**) Experimental design. (**B**) MRI taken at 38 weeks after Kras* activation shows normal pancreas morphology. P: pancreas, S: stomach, Sp: spleen. However, in the same animal, there is evidence of a small pancreatic tumor (T) at 40weeks, 2 days, which continues to increase in size over the next four weeks. (**C**) Tumor regression occurs following Kras* inactivation. By three weeks, there is no longer an identifiable tumor mass. (**D**) Tumor volume at the indicated time points. (**E**) Gross morphology of the pancreas following Kras* inactivation - note the small pancreas with no evident tumor mass (left panel). Histology of the regressed tissue (HE, middle panel, Scale bar 100 um) reveals acini (red arrowhead) surrounded by fibrosis (green arrowhead) and adipose tissue (blue arrowhead) with dilated ducts (yellow arrowhead) containing some cells that exhibit mucin accumulation identified by arrows (PAS staining, right panel, Scale bar 20 um). (**F**) Histology of fibrotic cysts, indicating a possible previous tumor site (HE, left panel), are lined with cells that are CK19 positive (inset). Scale bar 100 um. Gomori Trichrome (Scale bar 100 um), SMA staining (inset), p-ERK1/2, and Ki67 staining indicate that the remaining fibrosis is no longer reactive. Scale bars 20 um.

In order to determine the effect of Kras* inactivation on the primary tumor and metastases, we evaluated different possibilities for *in vivo* imaging, which would allow us to follow individual animals over time in longitudinal studies. Fluorodeoxyglucose positron emission tomography (FDG-PET) and magnetic resonance imaging (MRI) have been extensively used to image orthotopic models of PDA [20], [21], and, in some cases, for spontaneous tumors [22], [23]. Others have used high-resolution ultrasound in primary genetically engineered mouse models of PDA [6]. We explored the use of MRI, a clinically relevant imaging technique that would allow us to obtain high-resolution images of tumors and metastases and to measure volume changes over time. We imaged KPC mice (p48Cre; LSL-Kras; p53^R172H^) [4], in parallel with iKras*p53* mice to compare tumor formation in the two models. Initially, mice to be imaged were chosen based on clinical manifestation of disease (poor coat condition, distended abdomen), or, in some cases, upon palpation of an abdominal mass (**Fig. 3A** **)**. Control mice were imaged to visualize the normal pancreas, nested between the stomach, duodenum, spleen, and adjacent to the right kidney (**Fig. 3B**). In both individual KPC and iKras*p53* mice (**Fig. 3C and 3D**), MRI imaging clearly identified the pancreatic tumor mass. Additionally, in iKras*p53* mice, MRI also visualized multiple metastatic lesions to the liver, ranging from large to very small lesions (0.11 mm^3^) (**Fig. 3D** **,** bottom panels). In subsequent imaging experiments, the animals were imaged monthly, starting 1 month after activation of Kras* expression and induction of pancreatitis, and irrespective from any sign of disease. In this second cohort of animals, smaller tumors were occasionally identified so that tumor growth could be followed over time (**Fig. 3E** and **Fig. 4B**). Tumor and total metastases volumes were measured for individual animals (**Fig. 3F** **,** tumor volumes, top, and combined metastases volume, bottom, for KPC and iKras*p53* #1, #2, #3) at the indicated time points. Thus, this technique is an effective, non-invasive method to determine the presence of tumors and metastases in individual animals.

**Figure 5:**
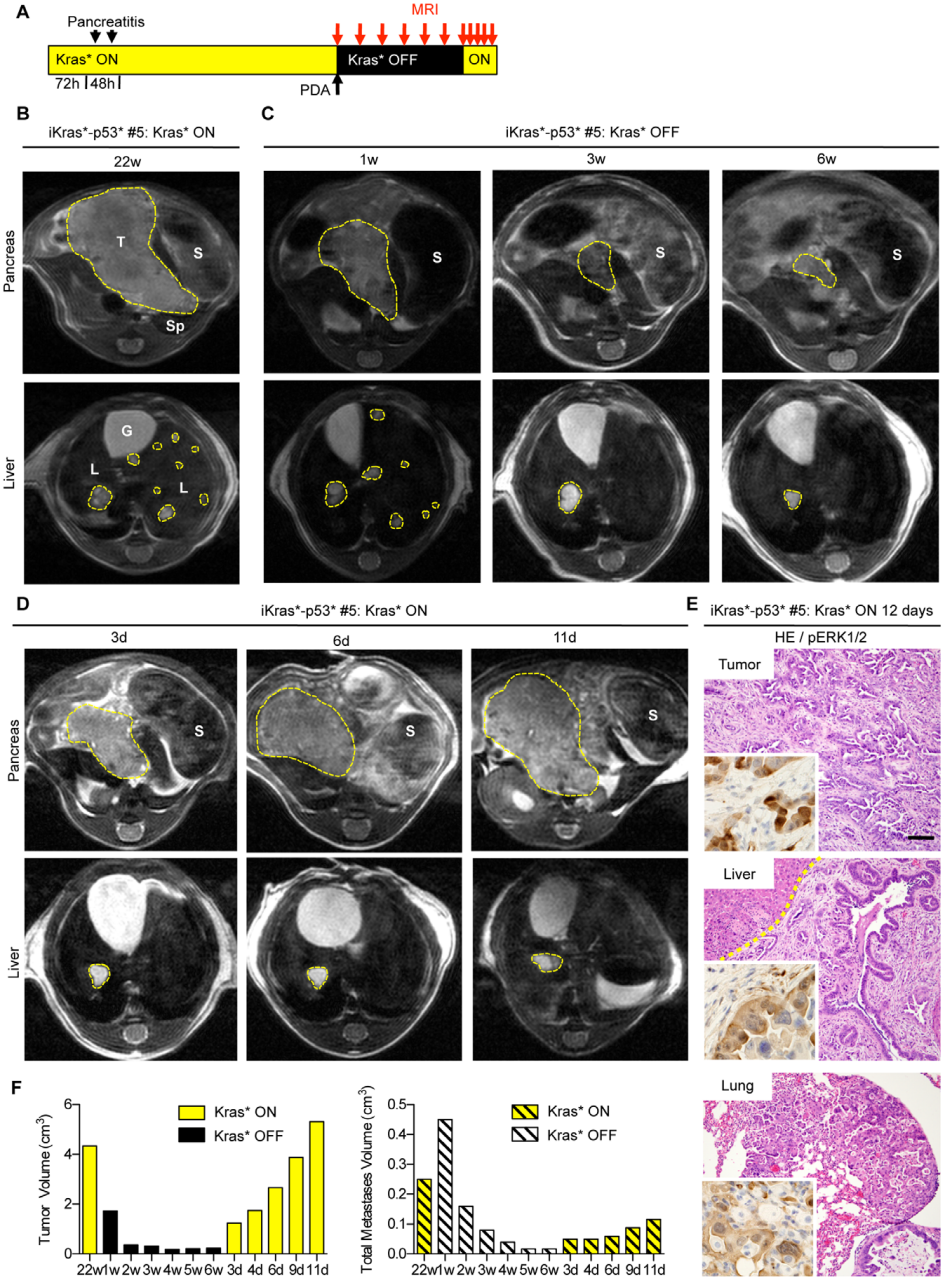
Pancreatic tumor relapse occurs following Kras* reactivation. (**A**) Experimental design. (**B**) Identification of a large pancreatic tumor mass 22 weeks after Kras* activation. T: tumor, S: stomach, Sp: spleen, L: liver, G: gallbladder. (**C**) Images taken 1, 3 and 6 weeks following Kras* inactivation. Note the greatly reduced primary tumor mass and metastatic load. (**D**) Reactivation of Kras* results in rapid tumor relapse. (**E**) Histology of the primary tumor as well as metastases found in the liver and lung following tumor relapse show abundant phospho-ERK1/2 expression (insets). Scale bar 100 um (**F**) Tumor and total metastases volume at the indicated time points.

In order to determine whether the primary tumor and metastases are dependent on Kras* we withdrew doxy in iKras*p53* mice, thus inactivating the Kras* transgene, and performed serial imaging of the same animal over time (scheme in **Fig. 4A** and **Fig. 5A**). Following Kras* inactivation, the primary tumor mass (**Fig. 4B** and **Fig. 5B**) regressed to barely detectable or undetectable within 3 weeks (**Fig. 4C** and **Fig. 5C**). By 6 weeks following Kras* inactivation, only rare metastatic lesions persisted, although with reduced size (**Fig. 5C** **)**. For each time point, we were able to obtain volumetric measures both of the primary tumor and of the metastases (**Fig. 4D** and **Fig. 5F**). When mice were dissected following prolonged Kras* inactivation, their pancreas appeared small and translucent, and lacked any apparent visible tumor mass (**Fig. 4E**). Histological analysis (**Fig. 4E**, middle panel) revealed fibrotic parenchyma (*green arrowhead*), with acini (*red arrowhead*) interspersed within dilated ducts (*yellow arrowhead*), and surrounded by adipose tissue (*blue arrowhead*). Some of the dilated ducts retained intracellular mucin accumulation identified by positive PAS staining (**Fig. 4E**, right panel). We also observed cysts lined with CK19-positive cells, that might indicate the previous tumor site (**Fig. 4F** **left panel**). The fibrotic areas retained collagen fibers, as highlighted with Trichrome staining, but the cells within them lacked Smooth Muscle Actin expression and were not proliferative, indicating scar tissue rather than active stroma. Both throughout the remaining pancreas and within the cysts, phospho-ERK1/2 expression was rare, confined to individual cells; Ki67 staining was present in a subset of the epithelial cells, but mitotic figures were rare (**Fig. 4F**). Thus, we concluded that in this spontaneous model of pancreatic cancer Kras* was required for the maintenance of both the primary tumor and metastases, even in the presence of an additional oncogene, mutant p53. Dependence of a single oncogene for advanced tumors has been observed before [24], [25], [26], [27], [28], [29]; however, to our knowledge, the effect of oncogene inactivation in metastases from solid tumors has been rarely addressed.

We next proceeded to determine whether the tumor cells had been completely eliminated, or whether a subset of them had survived inactivation of Kras*. For this purpose, we re-induced Kras* expression following tumor regression. Upon doxy administration, we observed rapid recurrence of the primary tumor mass (**Fig. 5D and 5F**), suggesting that some tumor cells had survived the transgene inactivation and were able to resume rapid growth. Additionally, further analysis revealed phospho-ERK1/2 levels were increased throughout the primary tumor as well as in the liver and lung metastases (**Fig. 5E**). This observation led us to investigate whether tumor cells might eventually acquire resistance to Kras* inactivation.

**Figure 6:**
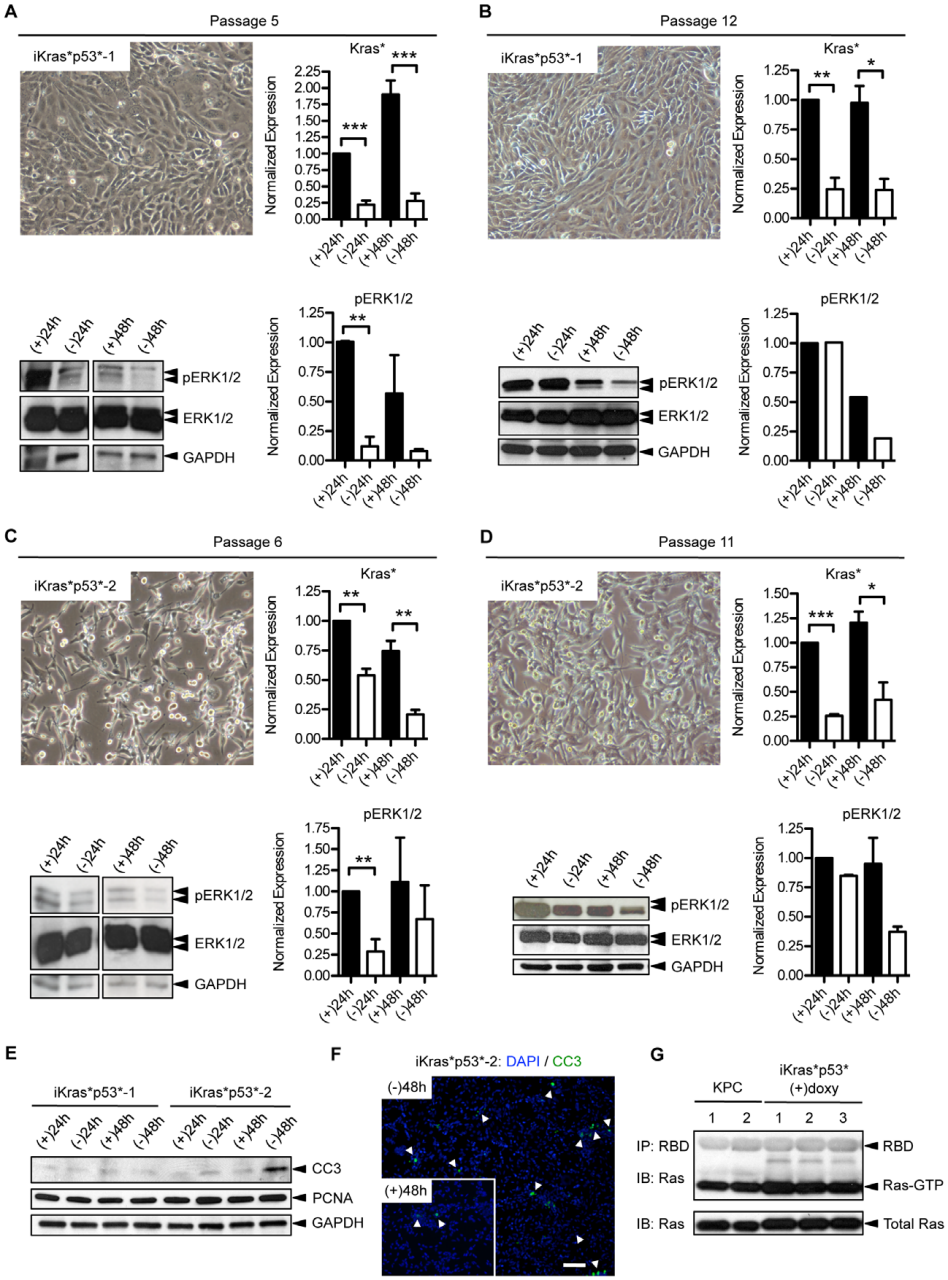
Characterization of primary pancreatic cancer cell lines from iKras*p53* mice. (**A**) Primary cell line iKras*p53*-1 exhibits epithelial morphology and demonstrates doxycycline dependent Kras* expression (* p<0.05), and pERK1/2 levels at passage 5. (**B**) The same cell line at passage 12; Kras* expression is still dependent on doxy (*** p<0.001), but pERK1/2 levels do not depend on Kras* expression. (**C**) A second primary cell line, iKras*p53*-2, has mesenchymal morphology. At passage 6, Kras* expression is dependent on doxy (** p<0.01), and pERK1/2 levels depend on Kras* expression. (**D**) Analysis at passage 11: Kras* is still regulated by doxy (* p<0.05, ** p<0.01), but pERK1/2 levels remain elevated. (**E**) Western blot analysis of apoptosis, indicated by cleaved caspase-3 (CC3), and proliferation, measured by proliferating cell nuclear antigen (PCNA), in both iKras*p53*-1 and iKras*p53*-2 cell lines. (**F**) Immunofluorescence of apoptosis, indicated by cleaved caspase-3 (CC3), in iKras*p53*-2 cells either in the presence of (+48 h) or absence (−48 h) of doxy in the media. DAPI staining marks the nuclei. Scale bar 100 um. (**G**) Ras pull-down assay demonstrates that Ras activity levels are comparable between iKras*p53* cell lines and cells from KPC tumors.

To address this possibility, and to be able to obtain histological information of the same tumor over time, we generated primary cell lines from iKras*p53* tumors. Primary tumor lines in culture were characterized either by epithelial or mesenchymal-like morphology (**Fig. 6A and 6C**). The expression of mutant Kras* RNA was regulated by doxy in culture, as expected (**Fig. 6A and 6C**). Furthermore, we determined Ras activity in the iKras*p53* primary cell lines was comparable with the levels of active Ras in cells extracted from KPC tumors (**Fig. 6G**). Phospho-ERK1/2 levels were initially regulated by doxy in the medium; however, this regulation was weakened over time in culture, with phospho-ERK1/2 levels becoming constitutively elevated, even though Kras* expression was still doxy-dependent (**Fig. 6B and 6D**). Interestingly, proliferation, as measured by proliferating cell nuclear antigen (PCNA), did not appear to be doxy-dependent in culture. However one of the cell lines (iKras*p53*-2) exhibited increased expression of the apoptosis marker cleaved caspase-3 in response to the removal of doxy from the media (**Fig. 6E and 6F**). The data is consistent with previous observations that a subset of human pancreatic cancer cells is dependent on oncogenic Kras for survival [35], [36].

**Figure 7:**
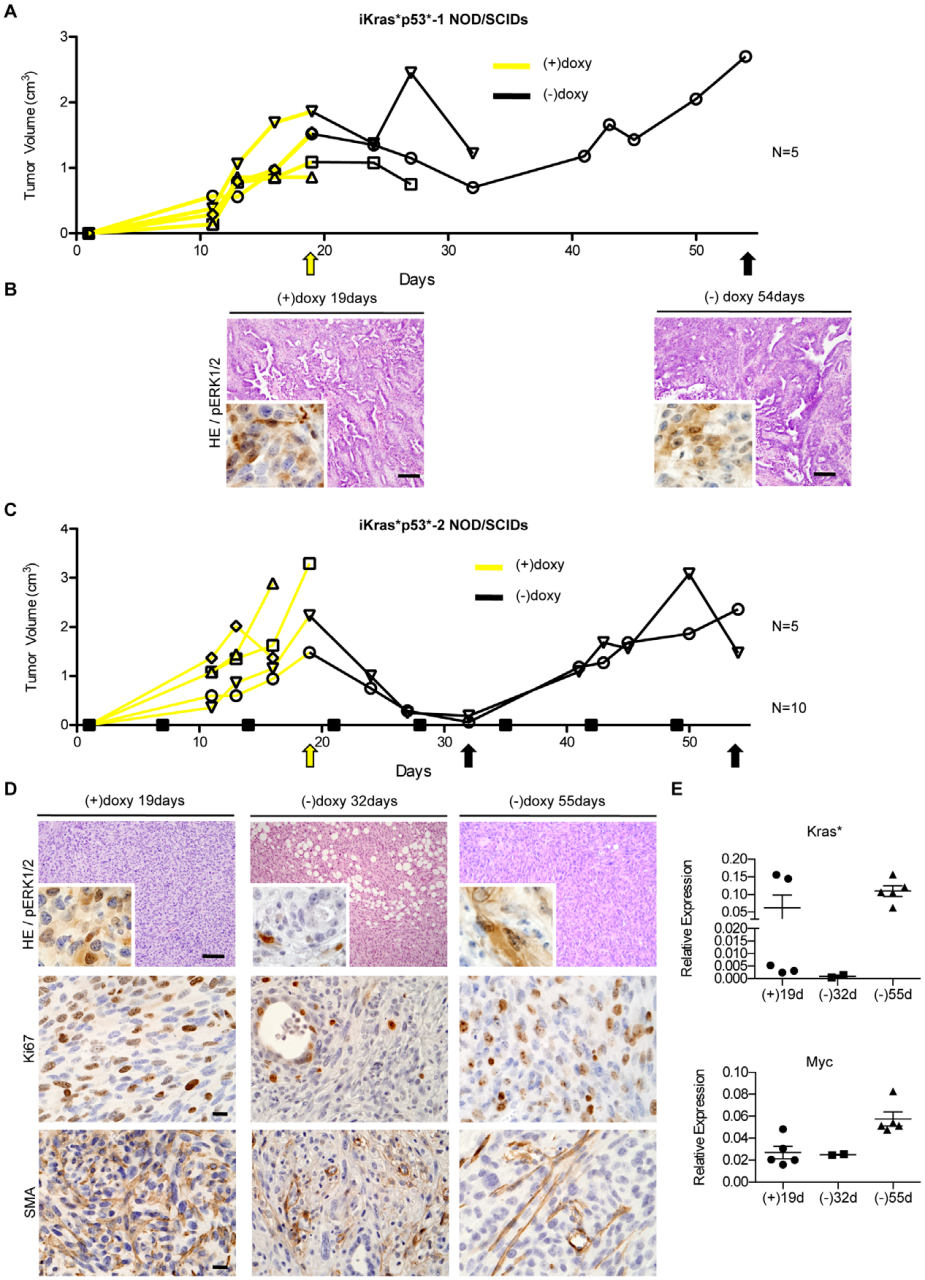
iKras*p53* cells reactivate Kras* expression independently of doxycycline regulation. (**A**) Tumor volume measured over time for iKras*p53*-1 cells transplanted subcutaneously in NOD/SCID mice. The yellow line indicates the presence of doxy, black lines indicate the absence of doxy, arrows indicate harvest time-points. N = 5. (**B**) Histology and phospho-ERK1/2 expression (inset) of iKras*p53*-1 tumors harvested during the initial growth phase and of relapsed tumors. Scale bar 100 um. (**C**) Tumor formation, regression, and relapse in NOD/SCID mice injected subcutaneously with primary cell line iKras*p53*-2. N = 5. A second cohort of NOD/SCID mice were injected with iKras*p53*-2 cells, but maintained in the absence of doxy which do not develop tumors. N = 10. The yellow line indicates the presence of doxy, black lines indicate the absence of doxy, arrows indicate harvest time-points. (**D**) Histology and pERK1/2 (inset), Ki67 and SMA expression of iKras*p53*-2 tumors during growth, regression, and relapse phases. Scale bar 100 um. (**E**) Quantitative PCR for oncogenic Kras* and myc expression in iKras*p53*-2 tumors.

To determine the effect of Kras* inactivation *in vivo*, we injected the cells subcutaneously in NOD/SCID mice. While subcutaneous injection is not appropriate for pre-clinical studies of pancreatic cancer, since it does not reflect the complexity of the tumor microenvironment, it nevertheless provides a readout of the ability of tumor cells to grow *in vivo*. All of the lines tested (n = 3) rapidly formed tumors when transplanted in NOD/SCID mice kept on doxy-water. Upon doxy withdrawal, the tumors first ceased growing and subsequently regressed rapidly; interestingly, complete regression was only observed in the cell line that was dependent on oncogenic Kras for survival in cell culture (iKras*p53*-2). After a latency period, however, the tumors grew back in the absence of doxy (**Fig. 7A** and **Fig. 7C**). Histological analysis showed that the transplanted tumors as well as the relapsed tumors resembled the primary tumor they were derived from (**Fig. 7B** and **Fig. 7D**, compare with **Fig. 1D**, top and bottom panels respectively). Both phospho-ERK1/2 and Ki67 expression levels were initially down-regulated upon Kras* inactivation (**Fig. 7D** **,** inset**)**, but expressed in the relapsed tumors (**Fig. 7B** and **Fig. 7D**). We also observed down-regulation of SMA in the fibroblasts surrounding the tumor cells, even though, as expected, SMA expression was retained in the vasculature-associated fibroblasts (**Fig. 7D**), indicating a change in epithelial-mesenchymal interactions upon Kras* inactivation in the epithelium. When we analyzed expression of oncogenic Kras* in the tumors, we found that the relapsed tumors had constitutively elevated expression of Kras*, indicating loss-of doxycycline dependence in the transgene (**Fig. 7E**). Thus, it appears likely that the tumor recurrence is due to selective pressure for Kras* expression, and not to acquired independence from Kras*. We also explored whether additional oncogenic pathways might contribute to the relapse. We did not detect any changes in the EGF signaling pathway, but we did observe an increase in myc expression in the relapsed tumors (**Fig. 7E**, data not shown).

In a different set of experiments, we injected tumor cells in NOD/SCID mice in absence doxy, to determine whether doxy-independent cells were already present in the tumor lines. In this case the tumor cells failed to give rise to tumors over a period of 13 weeks (**Fig. 7C**); thus, dysregulation of the Kras* transgene was not likely to be present in the initial cell population, but occurred while the cells were growing in NOD/SCID mice. In summary, our observations indicate that, *in vivo*, the tumor cells depend on oncogenic Kras* to form and maintain tumors. Of note, we did not observe recurrence in iKras*p53* mice, but only once the cells were cultured and re-established in NOD/SCID mice; it is therefore possible that the different environment, possibly because of the absence of a functional immune system**,** or because of the manipulation of the cells, might be permissive to tumor growth.

**Table 2:** Antibodies

Antibody	Supplier	Catalog Number	IHC dilution	Western dilution
CK19 (TromaIII)	Iowa Developmental Hybridoma Bank	–	1∶100	–
Gamma-H2AX	Millipore	05636	1∶400	–
Ki67	Vector Laboratories	VP-RM04	1∶100	–
P53	Cell Signaling	2524	1∶200	–
p-ERK1/2 (phospho-p44/42)	Cell Signaling	4370	1∶100	1∶1000
ERK1/2 (p44/42)	Cell Signaling	4695	–	1∶1000
Alpha-Smooth Muscle Actin	Sigma	A2547	1∶1000	–
PCNA	Thermo Scientific	RB9055P1	–	1∶500
Cleaved Caspase-3	Cell Signaling	9661	–	1∶1000
GAPDH	Abcam	Ab9485	–	1∶2500

## Discussion

Thanks to studies in mouse models that closely mimic the progression of the human disease, as well as genomic data and studies in primary human tumors, we have developed a sophisticated understanding of the biology of pancreatic cancer [1], [30], [31]. Given the complex mutational profile of pancreatic cancer and the impossibility to target every single alteration, it is of key importance to understand the genes/pathways that are required for tumor maintenance. Mutations in the Kras gene occur early during disease progression [32]. Mouse model studies have shown a key role of mutant Kras in the initiation of this disease [33], [34]. Moreover, a subset of human pancreatic cancer cell lines require Kras* for growth and survival both *in vitro* and in immuno-compromised host mice [35], [36]. Additionally, other human tumors such as lung adenocarcinoma and breast cancer show similar dependency on oncogenic Kras [17], [24]. We have recently shown that pancreatic cancer in the mouse is addicted to Kras* [8]. However, our first study did not extend to analyzing the role of Kras* in the presence of another oncogenic event, nor did it investigate metastatic disease. Given that pancreatic cancer is highly metastatic in humans, we felt it was essential to extend our approach to include this characteristic by combining Kras* expression with mutant p53*. Even in the presence of p53*, we show that Kras* is required for the maintenance of primary tumor and metastases. However, tumor cells survived Kras* inactivation and, upon Kras* reactivation, gave rise to renewed tumor growth. Moreover, when tumor cells were isolated and implanted in immuno-compromised hosts, they rapidly developed resistance. In other models addressing oncogene dependence, eventual acquisition of resistance has been commonly observed [24], [37]. In our system, it remains to be determined whether the presence of mutant p53 promotes resistance to Kras* inhibition, or whether the immuno-compromised status of the host is permissive for tumor relapse, as previously suggested [38]. Taken together, our findings validate the notion of inhibiting Kras in pancreatic cancer patients; however, they also provide a note of caution concerning the potential for tumor cells to eventually bypass their oncogene dependence. Future studies should be aimed at understanding the mechanisms that enable a subset of tumor cells to survive Kras* inactivation to provide strategies for complete tumor eradication.

## Materials and Methods

### Mice

Mice were housed in specific pathogen-free facilities of the University of Michigan Comprehensive Cancer Center. This study was approved by the University of Michigan University Committee on Use and Care of Animals (UCUCA) guidelines. p48Cre (Ptf1a-Cre) mice [14] were intercrossed with TetO-Kras^G12D^ [17], Rosa26^rtTa/rtTa^ [15] and p53^R172H/+^ [16] mice to generate p48Cre; TetO-Kras^G12D^; Rosa26^rtTa/+^; p53^R172H/+^ (iKras*p53*). Littermates lacking Cre or the mutant Kras and p53 alleles were used as controls. KPC mice [4] and iKras*p53^+/−^ mice [8] were previously described.

Doxy was administered through the drinking water, at a concentration of 0.2g/L in a solution of 5% sucrose, and replaced every 3–4 days.

Pancreatitis was induced through two series of eight hourly intraperitoneal injections of caerulein (Sigma), at a concentration of 75 ug/kg, over a 48-hour period, as previously described [18].

### Magnetic Resonance Imaging

Mice were anesthetized with 1–2% isoflurane/air, and body temperature was maintained by blowing warm air through the bore of the magnet using an Air-Therm (World Precision Instruments, Sarasota, FL). MRI scans were performed using a 7 T Agilent (Palo Alto, CA) *Direct Drive* system with a quadrature rat head volumic coil (m2m Imaging, Cleveland, OH). Mice were placed supine in the coil, taped below the thoracic cavity on the bed to reduce respiratory motion. T2-weighted images were acquired using a fast spin echo multi-slice sequence with TR/TE: 4000/30 ms, 8 echo trains, 4 averages, 2 dummy scans, field of view (FOV) = 25×25 mm^2^, matrix size = 128×128, slice thickness = 1 mm, number of slices = 25 contiguous. Using in-house software developed in MATLAB (The MathWorks, Inc., Natick, MA) the tumor boundary was manually defined on each slice and then integrated across slices to provide a volume estimate.

### Immunohistochemistry

Histology and Immunohistochemistry were performed as previously described [8]. A list of antibodies is provided in **Table 2** **.** Images were acquired with an Olympus BX-51 microscope, and Olympus DP71 digital camera, and DP Controller software.

### Establishment of Primary Cell Cultures

Tissue was harvested from the primary tumor, minced, and digested with 1 mg/ml collagenase V (Sigma) at 37°C for 15 minutes. Digestion was stopped with the addition of complete medium: RPMI-1640 (Gibco) +10% Fetal Bovine Serum +1% penicillin/streptomycin. Cells were isolated by filtration through a 100 um cell strainer and plated in complete medium containing doxycycline (Sigma) at 1 ug/ml.

### Subcutaneous Tumor Transplantation

1×10^6^ iKras*p53* cells were injected subcutaneously into the flank of NOD/SCID mice at a 1∶1 ratio of Matrigel (BD Biosciences) and complete medium. Doxy was administered through the drinking water at a concentration of 0.2 g/L in a solution of 5% sucrose for 3 days, and in chow (BioServ). Tumor size was measured by caliper.

### Quantitative RT-PCR

RNA extraction, cDNA preparation and quantitative PCR for Kras* and normalization to GAPDH was performed as previously described [8]. Statistical analysis was conducted with an unpaired t-test.

### Western Blot Analysis

Cells were lysed in RIPA buffer (SigmaAldrich, R0278) and protease inhibitor (Sigma-Aldrich, P8340). Equal amounts of protein were electrophoresed in 12% or 4–15% gradient SDS-PAGE gels, transferred to PVDF membrane (Bio-Rad). Membranes were blocked with 5% milk, and primary antibody incubations were performed overnight at 4°C. Primary antibodies and dilutions are provided in **Table 2**. Secondary antibody HRP-conjugated anti-rabbit (1∶5,000) was used and detected with Western Lightning Plus-ECL (Perkin Elmer). Protein bands were visualized on Kodak Biomax XAR film.

### Active Ras Pull-down Assay

Pull-down and immunoblotting of active Ras was performed using an Active Ras pull-down kit (Pierce) following the manufacture’s instructions.

## References

[1] Jones S, Zhang X, Parsons DW, Lin JC, Leary RJ, et al (2008) Core signaling pathways in human pancreatic cancers revealed by global genomic analyses. Science 321: 1801–1806. 18772397 10.1126/science.1164368 PMC2848990

[2] Hruban RH, Adsay NV, Albores-Saavedra J, Compton C, Garrett ES, et al (2001) Pancreatic intraepithelial neoplasia: a new nomenclature and classification system for pancreatic duct lesions. Am J Surg Pathol 25: 579–586. 11342768 10.1097/00000478-200105000-00003

[3] Petitjean A, Mathe E, Kato S, Ishioka C, Tavtigian SV, et al (2007) Impact of mutant p53 functional properties on TP53 mutation patterns and tumor phenotype: lessons from recent developments in the IARC TP53 database. Hum Mutat 28: 622–629. 17311302 10.1002/humu.20495

[4] Hingorani SR, Wang L, Multani AS, Combs C, Deramaudt TB, et al (2005) Trp53R172H and KrasG12D cooperate to promote chromosomal instability and widely metastatic pancreatic ductal adenocarcinoma in mice. Cancer Cell 7: 469–483. 15894267 10.1016/j.ccr.2005.04.023

[5] Olive KP, Tuveson DA (2006) The use of targeted mouse models for preclinical testing of novel cancer therapeutics. Clin Cancer Res 12: 5277–5287. 17000660 10.1158/1078-0432.CCR-06-0436

[6] Olive KP, Jacobetz MA, Davidson CJ, Gopinathan A, McIntyre D, et al (2009) Inhibition of Hedgehog signaling enhances delivery of chemotherapy in a mouse model of pancreatic cancer. Science 324: 1457–1461. 19460966 10.1126/science.1171362 PMC2998180

[7] Singh M, Lima A, Molina R, Hamilton P, Clermont AC, et al (2010) Assessing therapeutic responses in Kras mutant cancers using genetically engineered mouse models. Nat Biotechnol 28: 585–593. 20495549 10.1038/nbt.1640

[8] Collins MA, Bednar F, Zhang Y, Brisset JC, Galban S, et al (2012) Oncogenic Kras is required for both the initiation and maintenance of pancreatic cancer in mice. J Clin Invest 122: 639–653. 22232209 10.1172/JCI59227 PMC3266788

[9] Ying H, Kimmelman AC, Lyssiotis CA, Hua S, Chu GC, et al (2012) Oncogenic Kras maintains pancreatic tumors through regulation of anabolic glucose metabolism. Cell 149: 656–670. 22541435 10.1016/j.cell.2012.01.058 PMC3472002

[10] Morton JP, Timpson P, Karim SA, Ridgway RA, Athineos D, et al (2010) Mutant p53 drives metastasis and overcomes growth arrest/senescence in pancreatic cancer. Proc Natl Acad Sci U S A 107: 246–251. 20018721 10.1073/pnas.0908428107 PMC2806749

[11] Caulin C, Nguyen T, Lang GA, Goepfert TM, Brinkley BR, et al (2007) An inducible mouse model for skin cancer reveals distinct roles for gain- and loss-of-function p53 mutations. J Clin Invest 117: 1893–1901. 17607363 10.1172/JCI31721 PMC1904325

[12] Jackson EL, Olive KP, Tuveson DA, Bronson R, Crowley D, et al (2005) The differential effects of mutant p53 alleles on advanced murine lung cancer. Cancer Res 65: 10280–10288. 16288016 10.1158/0008-5472.CAN-05-2193

[13] Bardeesy N, Aguirre AJ, Chu GC, Cheng KH, Lopez LV, et al (2006) Both p16(Ink4a) and the p19(Arf)-p53 pathway constrain progression of pancreatic adenocarcinoma in the mouse. Proc Natl Acad Sci U S A 103: 5947–5952. 16585505 10.1073/pnas.0601273103 PMC1458678

[14] Kawaguchi Y, Cooper B, Gannon M, Ray M, MacDonald RJ, et al (2002) The role of the transcriptional regulator Ptf1a in converting intestinal to pancreatic progenitors. Nat Genet 32: 128–134. 12185368 10.1038/ng959

[15] Belteki G, Haigh J, Kabacs N, Haigh K, Sison K, et al (2005) Conditional and inducible transgene expression in mice through the combinatorial use of Cre-mediated recombination and tetracycline induction. Nucleic Acids Res 33: e51. 15784609 10.1093/nar/gni051 PMC1069131

[16] Olive KP, Tuveson DA, Ruhe ZC, Yin B, Willis NA, et al (2004) Mutant p53 gain of function in two mouse models of Li-Fraumeni syndrome. Cell 119: 847–860. 15607980 10.1016/j.cell.2004.11.004

[17] Fisher GH, Wellen SL, Klimstra D, Lenczowski JM, Tichelaar JW, et al (2001) Induction and apoptotic regression of lung adenocarcinomas by regulation of a K-Ras transgene in the presence and absence of tumor suppressor genes. Genes Dev 15: 3249–3262. 11751631 10.1101/gad.947701 PMC312852

[18] Morris JPt, Cano DA, Sekine S, Wang SC, Hebrok M (2010) Beta-catenin blocks Kras-dependent reprogramming of acini into pancreatic cancer precursor lesions in mice. J Clin Invest 120: 508–520. 20071774 10.1172/JCI40045 PMC2810083

[19] Fukuda A, Wang SC, Morris JPt, Folias AE, Liou A, et al (2011) Stat3 and MMP7 Contribute to Pancreatic Ductal Adenocarcinoma Initiation and Progression. Cancer Cell 19: 441–455. 21481787 10.1016/j.ccr.2011.03.002 PMC3075548

[20] He Z, Evelhoch JL, Mohammad RM, Adsay NV, Pettit GR, et al (2000) Magnetic resonance imaging to measure therapeutic response using an orthotopic model of human pancreatic cancer. Pancreas 21: 69–76. 10881935 10.1097/00006676-200007000-00054

[21] Grimm J, Potthast A, Wunder A, Moore A (2003) Magnetic resonance imaging of the pancreas and pancreatic tumors in a mouse orthotopic model of human cancer. Int J Cancer 106: 806–811. 12866043 10.1002/ijc.11281

[22] Abasolo I, Pujal J, Rabanal RM, Serafin A, Navarro P, et al (2009) FDG PET imaging of Ela1-myc mice reveals major biological differences between pancreatic acinar and ductal tumours. Eur J Nucl Med Mol Imaging 36: 1156–1166. 19252908 10.1007/s00259-009-1083-3

[23] Fendrich V, Schneider R, Maitra A, Jacobsen ID, Opfermann T, et al (2011) Detection of precursor lesions of pancreatic adenocarcinoma in PET-CT in a genetically engineered mouse model of pancreatic cancer. Neoplasia 13: 180–186. 21403843 10.1593/neo.10956 PMC3033596

[24] Podsypanina K, Politi K, Beverly LJ, Varmus HE (2008) Oncogene cooperation in tumor maintenance and tumor recurrence in mouse mammary tumors induced by Myc and mutant Kras. Proc Natl Acad Sci U S A 105: 5242–5247. 18356293 10.1073/pnas.0801197105 PMC2278195

[25] Chin L, Tam A, Pomerantz J, Wong M, Holash J, et al (1999) Essential role for oncogenic Ras in tumour maintenance. Nature 400: 468–472. 10440378 10.1038/22788

[26] D’Cruz CM, Gunther EJ, Boxer RB, Hartman JL, Sintasath L, et al (2001) c-MYC induces mammary tumorigenesis by means of a preferred pathway involving spontaneous Kras2 mutations. Nat Med 7: 235–239. 11175856 10.1038/84691

[27] Felsher DW, Bishop JM (1999) Reversible tumorigenesis by MYC in hematopoietic lineages. Mol Cell 4: 199–207. 10488335 10.1016/s1097-2765(00)80367-6

[28] Moody SE, Perez D, Pan TC, Sarkisian CJ, Portocarrero CP, et al (2005) The transcriptional repressor Snail promotes mammary tumor recurrence. Cancer Cell 8: 197–209. 16169465 10.1016/j.ccr.2005.07.009

[29] Debies MT, Gestl SA, Mathers JL, Mikse OR, Leonard TL, et al (2008) Tumor escape in a Wnt1-dependent mouse breast cancer model is enabled by p19Arf/p53 pathway lesions but not p16 Ink4a loss. J Clin Invest 118: 51–63. 18060046 10.1172/JCI33320 PMC2104482

[30] Morris JPt, Wang SC, Hebrok M (2010) KRAS, Hedgehog, Wnt and the twisted developmental biology of pancreatic ductal adenocarcinoma. Nat Rev Cancer 10: 683–695. 20814421 10.1038/nrc2899 PMC4085546

[31] Hezel AF, Kimmelman AC, Stanger BZ, Bardeesy N, Depinho RA (2006) Genetics and biology of pancreatic ductal adenocarcinoma. Genes Dev 20: 1218–1249. 16702400 10.1101/gad.1415606

[32] Kanda M, Matthaei H, Wu J, Hong SM, Yu J, et al.. (2012) Presence of Somatic Mutations in Most Early-Stage Pancreatic Intraepithelial Neoplasia. Gastroenterology. 10.1053/j.gastro.2011.12.042 PMC3321090 22226782

[33] Hingorani SR, Petricoin EF, Maitra A, Rajapakse V, King C, et al (2003) Preinvasive and invasive ductal pancreatic cancer and its early detection in the mouse. Cancer Cell 4: 437–450. 14706336 10.1016/s1535-6108(03)00309-x

[34] Aguirre AJ, Bardeesy N, Sinha M, Lopez L, Tuveson DA, et al (2003) Activated Kras and Ink4a/Arf deficiency cooperate to produce metastatic pancreatic ductal adenocarcinoma. Genes Dev 17: 3112–3126. 14681207 10.1101/gad.1158703 PMC305262

[35] Singh A, Greninger P, Rhodes D, Koopman L, Violette S, et al (2009) A gene expression signature associated with “K-Ras addiction” reveals regulators of EMT and tumor cell survival. Cancer Cell 15: 489–500. 19477428 10.1016/j.ccr.2009.03.022 PMC2743093

[36] Collisson EA, Sadanandam A, Olson P, Gibb WJ, Truitt M, et al (2011) Subtypes of pancreatic ductal adenocarcinoma and their differing responses to therapy. Nat Med 17: 500–503. 21460848 10.1038/nm.2344 PMC3755490

[37] Boxer RB, Jang JW, Sintasath L, Chodosh LA (2004) Lack of sustained regression of c-MYC-induced mammary adenocarcinomas following brief or prolonged MYC inactivation. Cancer Cell 6: 577–586. 15607962 10.1016/j.ccr.2004.10.013

[38] Rakhra K, Bachireddy P, Zabuawala T, Zeiser R, Xu L, et al (2010) CD4(+) T cells contribute to the remodeling of the microenvironment required for sustained tumor regression upon oncogene inactivation. Cancer Cell 18: 485–498. 21035406 10.1016/j.ccr.2010.10.002 PMC2991103

